# Anastrozole versus tamoxifen for the prevention of locoregional and contralateral breast cancer in postmenopausal women with locally excised ductal carcinoma in situ (IBIS-II DCIS): a double-blind, randomised controlled trial

**DOI:** 10.1016/S0140-6736(15)01129-0

**Published:** 2016-02-27

**Authors:** John F Forbes, Ivana Sestak, Anthony Howell, Bernardo Bonanni, Nigel Bundred, Christelle Levy, Gunter von Minckwitz, Wolfgang Eiermann, Patrick Neven, Michael Stierer, Chris Holcombe, Robert E Coleman, Louise Jones, Ian Ellis, Jack Cuzick, IBIS-II investigators

**Affiliations:** a Australia and New Zealand Breast Cancer Trials Group, University of Newcastle, Waratah, NSW, Australia; b Centre for Cancer Prevention, Wolfson Institute of Preventive Medicine, Queen Mary University of London, London, UK; c Barts Cancer Institute, Queen Mary University of London, London, UK; d Genesis Breast Cancer Prevention Centre, Manchester, UK; e Division of Cancer Prevention and Genetics, European Institute of Oncology, Milan, Italy; f South Manchester University Hospital, Manchester, UK; g Centre François Baclesse, Caen, France; h German Breast Group, Frankfurt, Germany; i Interdisziplinares Onkologisches Zentrum, Munich, Germany; j Department of Oncology, KU Leuven, University of Leuven, Leuven, Belgium; k Austrian Breast and Colorectal Cancer Study Group, Vienna, Austria; l Linda McCartney Centre, Royal Liverpool University Hospital, Liverpool, UK; m Department of Oncology and Metabolism, Weston Park Hospital, Sheffield, UK; n Department of Histopathology University of Nottingham, Nottingham, UK

## Abstract

**Background:**

Third-generation aromatase inhibitors are more effective than tamoxifen for preventing recurrence in postmenopausal women with hormone-receptor-positive invasive breast cancer. However, it is not known whether anastrozole is more effective than tamoxifen for women with hormone-receptor-positive ductal carcinoma in situ (DCIS). Here, we compare the efficacy of anastrozole with that of tamoxifen in postmenopausal women with hormone-receptor-positive DCIS.

**Methods:**

In a double-blind, multicentre, randomised placebo-controlled trial, we recruited women who had been diagnosed with locally excised, hormone-receptor-positive DCIS. Eligible women were randomly assigned in a 1:1 ratio by central computer allocation to receive 1 mg oral anastrozole or 20 mg oral tamoxifen every day for 5 years. Randomisation was stratified by major centre or hub and was done in blocks (six, eight, or ten). All trial personnel, participants, and clinicians were masked to treatment allocation and only the trial statistician had access to treatment allocation. The primary endpoint was all recurrence, including recurrent DCIS and new contralateral tumours. All analyses were done on a modified intention-to-treat basis (in all women who were randomised and did not revoke consent for their data to be included) and proportional hazard models were used to compute hazard ratios and corresponding confidence intervals. This trial is registered at the ISRCTN registry, number ISRCTN37546358.

**Results:**

Between March 3, 2003, and Feb 8, 2012, we enrolled 2980 postmenopausal women from 236 centres in 14 countries and randomly assigned them to receive anastrozole (1449 analysed) or tamoxifen (1489 analysed). Median follow-up was 7·2 years (IQR 5·6–8·9), and 144 breast cancer recurrences were recorded. We noted no statistically significant difference in overall recurrence (67 recurrences for anastrozole *vs* 77 for tamoxifen; HR 0·89 [95% CI 0·64–1·23]). The non-inferiority of anastrozole was established (upper 95% CI <1·25), but its superiority to tamoxifen was not (p=0·49). A total of 69 deaths were recorded (33 for anastrozole *vs* 36 for tamoxifen; HR 0·93 [95% CI 0·58–1·50], p=0·78), and no specific cause was more common in one group than the other. The number of women reporting any adverse event was similar between anastrozole (1323 women, 91%) and tamoxifen (1379 women, 93%); the side-effect profiles of the two drugs differed, with more fractures, musculoskeletal events, hypercholesterolaemia, and strokes with anastrozole and more muscle spasm, gynaecological cancers and symptoms, vasomotor symptoms, and deep vein thromboses with tamoxifen.

**Conclusions:**

No clear efficacy differences were seen between the two treatments. Anastrozole offers another treatment option for postmenopausal women with hormone-receptor-positive DCIS, which may be be more appropriate for some women with contraindications for tamoxifen. Longer follow-up will be necessary to fully evaluate treatment differences.

**Funding:**

Cancer Research UK, National Health and Medical Research Council Australia, Breast Cancer Research Fund, AstraZeneca, Sanofi Aventis.

## Introduction

Breast cancer is the most common cancer in women worldwide, with an estimated 1·6 million new cases reported every year.^1^ The proportion of these that are diagnosed as ductal carcinoma in situ (DCIS) has substantially increased over the past few decades due to the introduction of mammographic screening. It is estimated that approximately a fifth of all screen-detected breast cancers are DCIS.^2^

Management strategies for DCIS vary depending on histological grade, tumour characteristics, and extent of disease. Almost all aspects of treatment are controversial, including the need for any treatment for some screen-detected lesions,^3^ the extent of surgery,^4^ the use of radiotherapy,^5^, ^6^ and the use of adjuvant endocrine therapy.^7^, ^8^ The role of tamoxifen has been investigated in two large trials.^7^, ^8^ In the National Surgical Adjuvant Breast and Bowel Project (NSABP) B-24 trial,^7^ all women with DCIS received radiotherapy before being randomly assigned to tamoxifen or matching placebo. After a median of 6 years of follow-up, a significant 37% reduction in breast cancer recurrence was observed with tamoxifen compared with placebo.^7^ Retrospective evaluation of oestrogen receptors (ER) and progesterone receptors (PgR) in 732 patients from the original study showed that tamoxifen reduced subsequent breast cancer events by 51% for women with ER-positive DCIS.^9^ However, no significant benefit with tamoxifen was observed for women with ER-negative DCIS. In the UK/ANZ DCIS trial,^8^ 1578 women with locally excised DCIS were randomly assigned to receive tamoxifen with or without radiotherapy. After a median of 12·7 years of follow-up, tamoxifen significantly reduced all new breast cancer events by 29%, with a significant impact on ipsilateral DCIS recurrence and contralateral tumours, but no effect on ipsilateral invasive recurrence.^8^

**Research in Context**

**Evidence before this study**

A PubMed search between Jan 1, 1990, and Dec 31, 2002 (with the terms “ductal carcinoma in situ”, “breast cancer”, “aromatase inhibitors”, and “endocrine therapy”) and discussion with colleagues yielded no clinical trials or large cohorts of women with ductal carcinoma in situ (DCIS) treated by aromatase inhibitors. There have been two previous trials of tamoxifen. In the National Surgical Adjuvant Breast and Bowel Project (NSABP) B-24 trial, all women with DCIS received radiotherapy before being randomly assigned to tamoxifen or matching placebo. After a median of 6 years of follow-up, a significant 37% reduction in breast cancer recurrence was observed with tamoxifen compared with placebo. In the UK/ANZ DCIS trial, 1578 women with locally excised DCIS were randomly assigned to receive tamoxifen with or without radiotherapy. After a median of 12·7 years of follow-up, tamoxifen significantly reduced all new breast cancer events by 29%, with a significant effect on ipsilateral DCIS recurrence and contralateral tumours, but no effect on ipsilateral invasive recurrence. A further PubMed search was performed in October, 2015, which found no further published articles except the NSABP B-35 trial conference abstract.

**Added value of this study**

In combination with the B-35 trial, this trial provides the first evidence for the use of an aromatase inhibitor (here, anastrozole) compared with tamoxifen for postmenopausal women with locally excised hormone-receptor-positive DCIS after a median follow-up of 7·1 years. In this study, no clear efficacy differences were seen between the two treatments, although all available evidence supports a greater efficacy for anastrozole.

**Implications of all the available evidence**

Our results are consistent with the small benefit of anastrozole versus tamzifen as seen in the NSABP B-35 trial. This is also supported by direct evidence of greater efficacy for recurrence in adjuvant trials of women with early invasive cancer and indirect evidence of greater efficacy against new cancers in a preventive setting. Side-effect profiles between the drugs differed, but there was no clear overall advantage for either treatment. Anastrozole offers another treatment option for postmenopausal women with oestrogen-receptor-positive DCIS which might be more appropriate for some women with contraindications to tamoxifen.

Until now no data have been available on the use of aromatase inhibitors for DCIS. Two trials of very similar design have been conducted. Both compared anastrozole with tamoxifen in postmenopausal women with ER-positive or PgR-positive DCIS. The NSABP B-35 results will be reported elsewhere.^10^ Here, we report the first results from the International Breast Cancer Intervention Study-II DCIS (IBIS-II DCIS).

## Methods

### Study design and participants

We undertook a double-blind, randomised, placebo-controlled trial to compare anastrozole with tamoxifen for the prevention of locoregional and contralateral breast cancer. Participants were women aged 40–70 years, postmenopausal, and had DCIS diagnosed within 6 months before randomisation. Microinvasion of less than 1 mm was permitted. Patients treated by mastectomy were not eligible for this study but could be included in the IBIS-II breast cancer prevention trial.^11^ Radiotherapy was permitted according to local practice. Margin status was determined by the local pathologist and ER and PgR positivity was determined as greater than or equal to 5% positive cells (equivalent of Quick-score of three or above and H-score of ten or above). After a protocol amendment on Feb 24, 2009, women were also allowed to enter the trial if they had been diagnosed with atypical hyperplasia or lobular carcinoma in situ to allow treatment of these benign breast diseases known to respond to tamoxifen.^12^, ^13^

Exclusion criteria were: premenopausal at diagnosis; any previous diagnosis of breast cancer (including DCIS excised more than 6 months before randomisation or treated by mastectomy); diagnosis of any other cancer in the past 5 years (excluding non-melanoma skin cancer or in-situ cervical cancer); current treatment with anticoagulants; previous diagnosis of deep-vein thrombosis, transient ischaemic attack, or cerebrovascular accident; previous or current use of selective oestrogen receptor modulators; intention to use menopausal hormone therapy; unexplained postmenopausal bleeding; evidence of severe osteoporosis (T-score less than −4 at total hip or lumbar spine or more than two fragility fractures); history of lactose intolerance, glucose intolerance, or both; or life expectancy judged by the clinician to be less than 10 years.

All women provided written informed consent and the study was approved by the ethics committees of all participating institutions. The study sponsor was Queen Mary University of London.

### Randomisation and masking

Participants were randomly assigned in a 1:1 ratio to receive 1 mg/day oral anastrozole or 20 mg/day oral tamoxifen. Randomisation was stratified by major centre or hub. Randomised blocks (six, eight, or ten) were used to maintain balance and randomisation was performed centrally by electronic contact with the main trials centre. All treatment was given on a daily basis for 5 years and all women took two tablets per day (tamoxifen and anastrozole placebo, or anastrozole and tamoxifen placebo). All IBIS-II DCIS personnel, participants, and clinicians were masked to treatment allocation, except for the IBIS-II DCIS trial statistician, who had access to unblinded data, and the independent data monitoring committee, who reviewed interim data for safety purposes.

### Procedures

A dual-energy x-ray absorptiometry scan within 2 years before entry to the trial and two lateral spinal radiographs were required to assess bone density and vertebral fractures. Women were seen at 6 months, 12 months, and then annually up to the 5 year follow-up point at local clinics. Adherence to treatment was ascertained at each follow-up visit. After 5 years, follow-up was annual and either by a short postal questionnaire or clinic visit, depending on country. Clinical adverse events were recorded during the post-treatment follow-up period. Mammograms were performed at least every 2 years. Blood samples were taken at baseline, year 1, and year 5 for the evaluation of potential biomarkers.

### Outcomes

The primary endpoint of this analysis was the development of histologically confirmed breast cancer, both invasive and new or recurrent DCIS. First events were further categorised as local recurrence (all ipsilateral disease), distant recurrence (including node-positive contralateral disease and recurrences at distant sites [eg, lung, bone, etc]), or isolated contralateral events. Secondary endpoints included ER status, breast cancer mortality, other cancers, cardiovascular disease, fractures, adverse events, and non-breast cancer deaths. Prespecified subgroup analyses of recurrence were for invasive versus DCIS, contralateral versus ipsilateral, and ER status (ER positive *vs* ER negative); other subgroup analyses were exploratory. Further post-hoc analyses included PgR and HER2 receptor status for invasive recurrence only. Future plans are to explore outcomes by ER levels and HER2 status of the primary tumour when tissue collection is complete, and to examine timing effects of treatment after the initial 5 year treatment period is completed.

### Statistical analysis

All analyses were done on a modified intention-to-treat basis, including all women who were enrolled, randomly assigned, and did not revoke consent for use of their data. Analyses of the efficacy endpoints were based on hazard ratios (HRs). Cox proportional hazard models^14^, ^15^ were used to derive these with corresponding 95% CIs. The analysis plan first tested non-inferiority of anastrozole (upper 95% CI of HR <1·25) and, if successful, then for the superiority of anastrozole. Survival curves were estimated using the Kaplan-Meier method.^16^ Secondary endpoints were compared using odds ratios (ORs), which closely approximate relative risk for rare events. Adverse events are presented if predefined or occurred in at least 5% of participants, and Fisher's exact tests were used to compare adverse events when appropriate. Adherence was calculated using the Kaplan-Meier method, censoring at breast cancer recurrence, death, or 5 years of follow-up. All p values were two-sided.

We estimated a required sample size of 4000 on the basis of a 1·6% annual recurrence rate for tamoxifen-treated patients with a 16·7% relative reduction for anastrozole to show non-inferiority, and a 33% reduction to show superiority with 5-year median follow-up. Recruitment to the trial closed on Feb 8, 2012, after enrolment of 2980 of the 4000 planned participants. During the course of the trial, local recurrences occurred at less than half the rate anticipated in the analysis plan, due largely to improvements in the surgical treatment of DCIS. Consequently, the required numbers of events anticipated in the protocol would not be reached for a number of years, and the IBIS-II steering committee, with the agreement of the independent data monitoring committee, took the decision to analyse and report the results at this stage; data were collected up to the cutoff date of Sept 30, 2015.

All analyses were done using Stata version 13.1. This trial is registered at the ISRCTN registry, number ISRCTN37546358.

### Role of the funding source

The study funders had no role in design, data collection, data analysis, data interpretation, or writing of the report. IS had full access to all data in the study, and JC, JFF, and AH had final responsibility for the decision to submit for publication.

## Results

**Figure 1:**
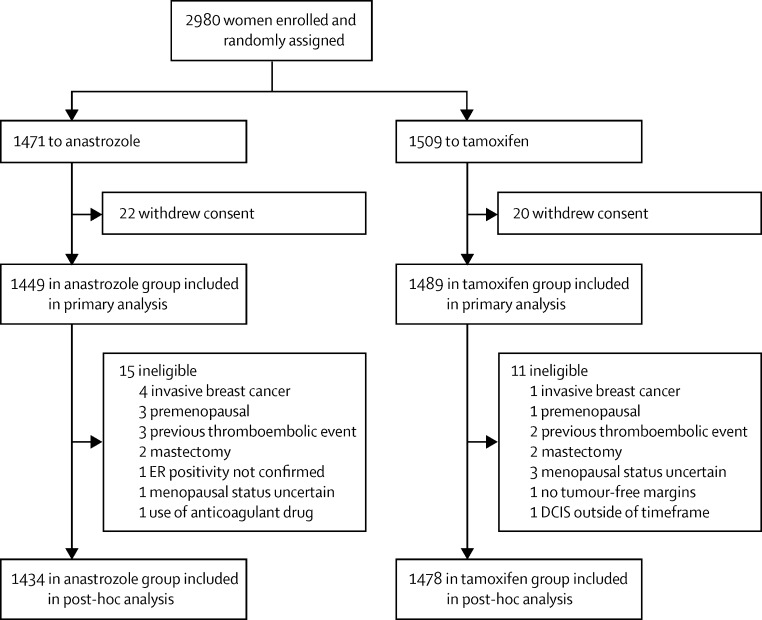
Trial profile ER=oestrogen receptor. DCIS=ductal carcinoma in situ.

Between March 6, 2003, and Feb 8, 2012, we recruited 2980 postmenopausal women with locally excised ER-positive or PgR-positive DCIS in 236 centres from 14 countries, and randomly assigned them to receive anastrozole (n=1471) or tamoxifen (n=1509; figure 1). A total of 42 women (22 in the anastrozole group, 20 in the tamoxifen group) withdrew their consent to use their data, leaving 2938 women for the primary analysis (figure 1). Baseline characteristics are presented in table 1. A further 26 women were found to be ineligible after randomisation (figure 1) but were included in the primary analysis. Median age was 60·3 years (IQR 56·1–64·6), and 658 (22%) were older than 65 years. Median body-mass index was 26·7 kg/m^2^ (IQR 23·6–30·4), with 903 (31%) of women being obese (>30 kg/m^2^) at baseline (table 1). Median age at menarche was 13 years (IQR 12–14) and at birth of first child was 24 years (IQR 21–27), and 814 (28%) women had had a hysterectomy before trial entry. 1336 (45%) women had used menopausal hormone therapy before trial entry and two-thirds were never-smokers (table 1). Only nine women (<1%) with atypical hyperplasia or lobular carcinoma in situ were entered into the trial.

Baseline DCIS tumour characteristics are also shown in table 1. Median DCIS major diameter was 13 mm (IQR 7–22), median clear margin distance was 5 mm (IQR 2–10), and most women had either intermediate-grade (1224; 42%) or high-grade (1129; 38%) tumours. Radiotherapy was given to 2091 (71%) women. Again, we noted no significant differences between treatment groups.

The cutoff date for this analysis was Sept 30, 2015. Median follow-up was 7·2 years (IQR 5·6–8·9) and 21 112 women-years of follow-up were accrued (10 670 women-years for anastrozole and 10 442 tamoxifen). 5 year adherence was estimated to be 67·6% (95% CI 65·1–70·0) in the anastrozole group compared with 67·4% (64·9–69·7) in the tamoxifen group (p=0·71; appendix). The main reasons for treatment cessation were adverse events and patient decision (data not shown).

**Figure 2:**
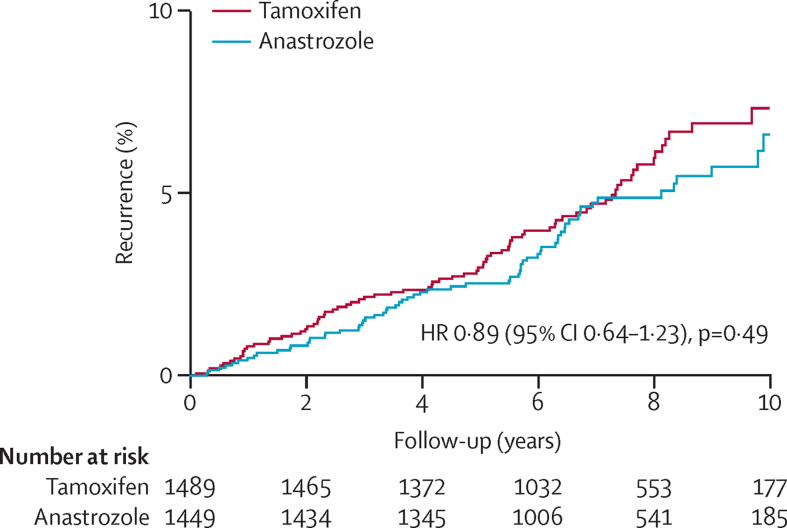
Recurrence for all breast cancer according to treatment allocation

A total of 144 breast cancer recurrences were reported; recurrences were mostly invasive (84 [58%]; table 2). Numerically fewer recurrences occurred with anastrozole (67 recurrences; annual rate 0·64% [95% CI 0·50–0·82]) than for tamoxifen (77; 0·72% [0·58–0·90]; HR 0·89 [95% CI 0·64–1·23]; figure 2). The non-inferiority of anastrozole was established (upper 95% CI <1·25), but its superiority to tamoxifen was not (p=0·49). Kaplan-Meier estimates of recurrence at 5 years were 2·5% (95% CI 1·8–3·5) for anastrozole and 3·0% (2·2–4·0) for tamoxifen. After 10 years of follow-up, recurrence was 6·6% (95% CI 4·9–8·8) and 7·3% (5·7–9·4), respectively.

Among the 144 recurrences, 86 (60%) were ER-positive, 30 (21%) were ER-negative, and ER status was missing for 28 (19%). Among women with ER-positive recurrences, 30 (2%) were in the anastrozole group compared with 56 (4%) in the tamoxifen group (HR 0·55 [95% CI 0·35–0·86], p=0·008). Among women with ER-negative recurrences, 17 (1%) were in the anastrozole group compared with 13 (<1%) in the tamoxifen group (HR 1·34 [95% CI 0·65–2·75], p=0·43).

Analyses adjusted by age, body-mass index, menopausal hormone therapy use, grade, margins, and radiotherapy subgroups yielded similar HRs as in the univariate analyses (table 2). Similar numbers of DCIS recurrences were observed in each treatment group (29 for anastrozole *vs* 30 for tamoxifen; HR 0·99 [95% CI 0·60–1·65], p=0·98; table 2).

A total of 69 deaths had been reported by the cutoff date (appendix). Overall, we noted no statistically significant difference between treatment arms (33 for anastrozole *vs* 36 for tamoxifen; HR 0·93 [95% CI 0·58–1·50], p=0·78) and no specific cause of death differed by treatment group. Only four deaths from breast cancer were recorded, one in the anastrozole group and three in the tamoxifen group. Overall, the frequency of cancers other than breast was not significantly different in the anastrozole and tamoxifen groups (61 *vs* 71; OR 0·88 [95% CI 0·61–1·26], p=0·47; table 3). However, endometrial, ovarian, and skin cancers were significantly more common with tamoxifen (table 2).

We collected a comprehensive record of side-effects during the 5 years of treatment (table 4). The number of women reporting any event was similar between treatment groups overall (1323 for anastrozole *vs* 1379 for tamoxifen) but the specific profiles were different. Fractures were significantly higher in the anastrozole group (129 *vs* 100; OR 1·36 [95% CI 1·03–1·80], p=0·027) and musculoskeletal adverse events such as joint stiffness, paraesthesia, carpal tunnel syndrome, and osteoporosis were also significantly higher with anastrozole (table 4). Hypercholesterolaemia was furthermore significantly more common in women receiving anastrozole compared with those receiving tamoxifen, probably as a result of the cholesterol-reducing effects of tamoxifen. By contrast, anastrozole was associated with substantially fewer muscle spasms compared with tamoxifen (25 [2%] *vs* 106 [7%]; OR 0·23 [95% CI 0·14–0·36], p<0·0001).

Apart from vaginal dryness, gynaecological symptoms were significantly higher with tamoxifen. Vasomotor symptoms were common in both treatment groups, but the frequency was significantly lower with anastrozole (818 [56%] *vs* 899 [60%]; OR 0·85 [95% CI 0·73–0·99], p=0·0310; table 4). We noted a significant decrease in pulmonary emboli and deep vein thromboses (seven *vs* 24; OR 0·30 [95% CI 0·11–0·71], p=0·0028). No statistically significant difference was seen for cardiovascular events overall or myocardial infarction in particular (table 4). However, transient ischaemic attacks (13 *vs* five; OR 2·69 [95% CI 0·90–9·65], p=0·05) and particularly cerebrovascular accidents (13 *vs* four; 3·36 [1·04–14·18], 0·025) were increased with anastrozole.

Despite the differences in side-effect profiles, treatment adherence was virtually identical between treatment groups and was 67·6% for anastrozole and 67·4% for tamoxifen after 5 years (appendix).

**Figure 3:**
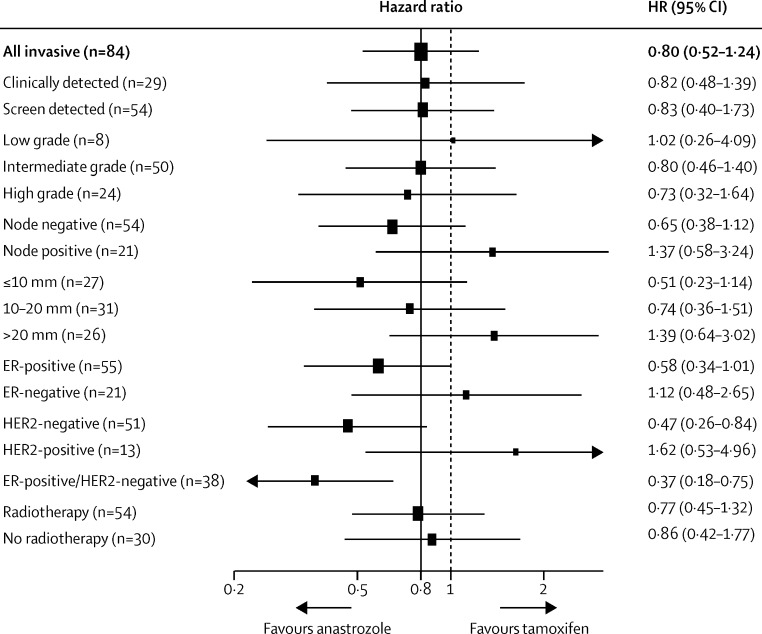
Subgroup analyses for invasive breast cancer by cancer characteristics Numbers do not add to totals because of missing values. The dotted line shows no effect point, and the bold line shows overall treatment effect point. ER=oestrogen receptor.

In a post-hoc analysis, we assessed differences by subgroups of tumour for invasive recurrence (figure 3). The largest difference was noted for invasive ER-positive/HER2-negative tumours (10 recurrences with anastrozole *vs* 28 with tamoxifen; HR 0·37 [95% CI 0·18–0·75], p=0·0060; figure 3). HER2-positive tumours showed better efficacy with tamoxifen (HR 1·62 [95% CI 0·53–4·96]; heterogeneity p=0·05; figure 3).

We did not find a differential effect on recurrence according to radiotherapy use at baseline (54 recurrences with radiotherapy *vs* 30 with no radiotherapy; HR 0·77 [95% CI 0·49–1·21], p=0·25). Furthermore, anastrozole was not more effective at reducing invasive recurrences in those women who had radiotherapy at baseline (HR 0·77 [95% CI 0·45–1·32], p=0·34) compared with those who did not (0·86 [0·42–1·77], p=0·69; heterogeneity p=0·79; figure 3).

In a post-hoc analysis, we excluded 26 women who were found to be ineligible after randomisation (figure 1). Exclusion of these women from the primary analysis did not alter the results (data not shown). Furthermore, exclusions of the nine (<1%) women with atypical hyperplasia or lobular carcinoma in-situ from a post-hoc reassessment of the primary endpoint analysis had no effect on the results (data not shown).

## Discussion

In this large, randomised, double-blind, placebo-controlled trial comparing anastrozole with tamoxifen in women with ER-positive or PgR-positive DCIS treated by wide local excision with or without breast radiotherapy, the non-inferiority of anastrozole to tamoxifen was demonstrated, but a significant superiority efficacy was not, although we noted a slightly lower recurrence rate for anastrozole. However, the overall event rate was lower than anticipated, which might have contributed to non-significant results with wide confidence intervals, and as a result smaller effects of anastrozole might have been missed. This possible small benefit for anastrozole is consistent with the larger 27% reduction seen in the similar NSABP B-35 trial, which was statistically significant (p=0·03).^10^ Trials in the adjuvant setting have also indicated greater efficacy for anastrozole and other aromatase inhibitors versus tamoxifen.^17^, ^18^ Additionally, the reduction in contralateral breast cancer is consistent with the benefits of anastrozole compared with tamoxifen seen in the ATAC trial^17^ and compared with placebo in the IBIS-II breast cancer prevention trial.^11^ The greater efficacy of anastrozole for ER-positive or HER2-negative invasive recurrence has been seen elsewhere for invasive disease.^19^ HER2 status was not routinely collected for the baseline tumour, but tumour blocks are being collected retrospectively and its impact along with other markers will be reported at a later stage. Local recurrence rates were lower than those predicted on the basis of earlier trials. This is probably caused by greater attention to achieving clear surgical margins, and improvements in and more frequent use of radiotherapy.

Clear differences were seen in the side-effect profile. Many side-effects followed the expected pattern seen during treatment of invasive cancer,^18^ with a higher fracture rate and more musculoskeletal events with anastrozole, and more venous thromboembolic events, gynaecological events, and vasomotor symptoms with tamoxifen. The higher rate of strokes with anastrozole is surprising because this pattern was not seen in ATAC (62 strokes with anastrozole *vs* 80 with tamoxifen),^17^ or the prevention component of IBIS-II (three with anastrozole *vs* six with placebo),^11^ and these events were not lower for tamoxifen when compared with placebo in IBIS-I (ten with tamoxifen *vs* 12 with placebo).^20^ Tamoxifen has previously been reported to reduce headache occurrence,^20^, ^21^ so the higher rate of headache in the anastrozole group of our trial probably resulted from this rather than the effects of anastrozole. However, increased hypertension was seen for anastrozole in both ATAC^17^ and IBIS-II prevention;^11^ the small increase in our anastrozole group therefore seems to be a real treatment effect, although the mechanism is not understood.

Although occurrence of other cancers was similar overall, the incidence of specific cancers differed by treatment. A two-to-three-fold increase in endometrial cancer is well documented for tamoxifen,^22^, ^23^ by contrast with a reduced incidence compared with the general population anticipated for anastrozole in view of the strong hormone dependence for this tumour.^24^ In combination, these two effects account for the striking difference seen here. Ovarian cancer is not known to be affected by tamoxifen and the differences here probably result from a preventive effect of anastrozole, as previously seen in IBIS-II prevention (four cases with anastrozole *vs* seven with placebo) and indirectly in ATAC (ten with anastrozole *vs* 17 with tamoxifen), and supported by the increased risk associated with use of menopausal hormone therapy.^25^ A decrease of colorectal cancer has been reported in users of menopausal hormone therapy;^26^ although a small increase was reported in ATAC (39 cases with anastrozole *vs* 31 with tamoxifen), a lower risk was seen in IBIS-II prevention (three with anastrozole *vs* 11 with tamoxifen), so the role of aromatase inhibitors in affecting risk of colorectal cancer remains uncertain.

The major strengths of this study include its multinational nature, large size, moderate length of follow-up, and detailed collection of side-effect data. The major limitation of this trial was the lower-than-expected event rate, which adds uncertainty about the lack of significance of some of the small differences seen. A few unexpected side-effects were also recorded, which require further validation in view of the amount of multiple testing. It is too early to assess the effect of these treatments on mortality and long-term follow-up; a full meta-analysis of all major endpoints with the B-35 study is planned to study these issues.

In summary, anastrozole offers another option for postmenopausal women with ER-positive DCIS, and the choice between it and tamoxifen will probably depend more on previous history of other conditions (eg, osteoporosis and venous thrombosis) and short-term tolerability (musculoskeletal, vasomotor, and gynaecological symptoms) than differences in efficacy.

## Figures and Tables

**Table 1:** Baseline demographic and tumour characteristics according to treatment allocation

		**Anastrozole (n=1449)**	**Tamoxifen (n=1489)**
Age, years		60·4 (56·4–64·5)	60·3 (55·8–64·5)
BMI, kg/m^2^		26·7 (23·5–30·4)	26·7 (23·7–30·2)
Age at menarche, years		13·0 (12·0–14·0)	13·0 (12·0–14·0)
Age at birth of first child		24·0 (21·0–27·0)	24·0 (21·0–27·0)
Smoking			
	Never	890 (61%)	934 (63%)
	Ever	496 (34%)	495 (33%)
	Missing	63 (4%)	60 (4%)
Menopausal hormone therapy use		678 (47%)	658 (44%)
Hysterectomy		406 (21%)	408 (22%)
Radiotherapy		1027 (71%)	1064 (71%)
Tumour size, mm		13 (7–22)	13 (7–22)
Margins, mm		5 (2–10)	5 (2–10)
Grade			
	Low	293 (20%)	279 (19%)
	Intermediate	606 (42%)	618 (42%)
	High	542 (37%)	587 (39%)
	Missing	8 (<1%)	5 (<1%)
Laterality			
	Left	742 (51%)	789 (53%)
	Right	703 (49%)	696 (47%)
	Bilateral	5 (<1%)	4 (<1%)

Data are median (IQR) or n (%). Characteristics given for the modified intention-to-treat population; numbers of individuals do not add to totals because of missing values. BMI=body-mass index.

**Table 2:** All breast cancer, invasive, and DCIS recurrences according to treatment allocation

		**Anastrozole (n=1449)**	**Tamoxifen (n=1489)**	**Unadjusted analysis**		Adjusted analysis*	
				HR (95% CI)	p value	HR (95% CI)	p value
All		67 (5%)	77 (5%)	0·89 (0·64–1·23)	0·49	0·83 (0·59–1·18)	0·31
Invasive†		37 (3%)	47 (3%)	0·80 (0·52–1·24)	0·32	0·72 (0·46–1·14)	0·16
	Ipsilateral	20 (1%)	22 (1%)	0·93 (0·51–1·71)	0·82	0·77 (0·40–1·48)	0·44
	Contralateral	17 (1%)	25 (2%)	0·69 (0·37–1·28)	0·24	0·68 (0·36–1·29)	0·24
DCIS		29 (2%)	30‡ (2%)	0·99 (0·60–1·65)	0·98	0·98 (0·57–1·69)	0·95
	Ipsilateral	21 (1%)	23 (2%)	0·94 (0·52–1·69)	0·83	1·03 (0·55–1·91)	0·93
	Contralateral	8 (<1%)	6 (<1%)	1·37 (0·47–3·94)	0·56	1·02 (0·33–3·18)	0·97

DCIS=ductal carcinoma in situ. HR=hazard ratio.

* Adjusted for age, body-mass index, menopausal hormone therapy, grade, margins, and radiotherapy.

† 1 missing for invasiveness.

‡ 1 missing data for laterality.

**Table 3:** Frequency of cancers other than breast according to treatment allocation

		**Anastrozole (n=1449)**	**Tamoxifen (n=1489)**	**OR (95% CI)**	**p value**
Total		61	71	0·88 (0·61–1·26)	0·47
Gynaecological		1	17*	0·06 (0·001–0·386)	0·0002
	Endometrial	1	11	0·09 (0·002–0·64)	0·0044
	Ovarian	0	5	0·00 (0·00–0·79)	0·027
Lung		11	7	1·62 (0·57–4·94)	0·32
Gastrointestinal		16	10	1·65 (0·70–4·08)	0·21
	Colorectal	10	5	2·06 (0·64–7·71)	0·18
Lymphoma or leukaemia		8	5	1·65 (0·47–6·42)	0·44
Skin		12	23	0·53 (0·24–1·12)	0·07
	Melanoma	4	4	1·03 (0·19–5·53)	0·97
	Non-melanoma	8	19	0·43 (0·16–1·03)	0·040
Other		13	9	1·49 (0·59–3·96)	0·36

OR=odds ratio.

* One cervical cancer.

**Table 4:** Adverse events reported at any time according to treatment allocation

		**Anastrozole (n=1449)**	**Tamoxifen (n=1489)**	**OR (95% CI)**	**p value**
Fractures		129 (9%)	100 (7%)	1·36 (1·03–1·80)	0·027
	Pelvic or hip	11 (1%)	4 (<1%)	2·84 (0·84–12·25)	0·06
	Spine	6 (<1%)	6 (<1%)	1·03 (0·27–3·85)	0·96
Musculoskeletal (any)		929 (64%)	811 (54%)	1·49 (1·28–1·74)	<0·0001
	Arthralgia	832 (57%)	729 (49%)	1·41 (1·21–1·63)	<0·0001
	Joint stiffness	74 (5%)	35 (2%)	2·24 (1·46–3·47)	<0·0001
	Paraesthesia	42 (3%)	23 (2%)	1·90 (1·11–3·33)	0·013
	Carpal tunnel syndrome	35 (2%)	11 (1%)	3·33 (1·64–7·29)	<0·0001
	Osteoporosis	97 (7%)	54 (4%)	1·91 (1·34–2·73)	<0·0001
	Muscle spasm	25 (2%)	106 (7%)	0·23 (0·14–0·36)	<0·0001
Vasomotor or gynaecological (any)		879 (61%)	1031 (69%)	0·69 (0·59–0·80)	<0.0001
	Hot flushes	818 (56%)	899 (60%)	0·85 (0·73–0·99)	0·031
	Vaginal dryness	189 (13%)	159 (11%)	1·25 (1·00–1·58)	0·047
	Vaginal haemorrhage	35 (2%)	80 (5%)	0·44 (0·28–0·66)	<0·0001
	Vaginal discharge	30 (2%)	136 (9%)	0·21 (0·14–0·32)	<0·0001
	Vaginal candidiasis	8 (1%)	42 (3%)	0·19 (0·08–0·41)	<0·0001
Other					
	Headache	82 (6%)	61 (4%)	1·40 (0·99–2·00)	0·049
	Hypercholesterolaemia	43 (3%)	11 (1%)	4·11 (2·07–8·86)	<0·0001
Major thromboembolic		7 (<1%)	24 (2%)	0·30 (0·11–0·71)	0·0028
	Pulmonary embolism	5 (<1%)	8 (1%)	0·64 (0·16–2·23)	0·43
	Deep vein thrombosis (without pulmonary embolism)	2 (<1%)	16 (1%)	0·13 (0·01–0·54)	0·0011
Any cardiovascular		93 (6%)	84 (6%)	1·15 (0·84–1·57)	0·38
	Myocardial infarction	6 (<1%)	6 (<1%)	1·03 (0·27–3·85)	0·99
	Cerebrovascular accident	13 (1%)	4 (<1%)	3·36 (1·04–14·18)	0·025
	Transient ischaemic attack	13 (1%)	5 (<1%)	2·69 (0·90–9·65)	0·05
	Hypertension	82 (6%)	73 (5%)	1·16 (0·83–1·63)	0·36
Any eye disease		230 (16%	209 (14%)	1·16 (0·94–1·42)	0·16
	Cataract	72 (5%)	61 (4%)	1·22 (0·85–1·77)	0·26

OR=odds ratio.

## References

[1] Jemal A, Bray F, Center MM (2011). Global cancer statistics. CA Cancer J Clin.

[2] Mokbel K, Cutuli B (2006). Heterogeneity of ductal carcinoma in situ and its effects on management. Lancet Oncol.

[3] Francis A, Thomas J, Fallowfield L (2015). Addressing overtreatment of screen detected DCIS: the LORIS trial. Eur J Cancer.

[4] Worni M, Akushevich I, Greenup R (2015). Trends in treatment patterns and outcomes for ductal carcinoma in situ. J Natl Cancer Inst.

[5] Goodwin A, Parker S, Ghersi D, Wilcken N (2013). Post-operative radiotherapy for ductal carcinoma in situ of the breast. Cochrane Database Syst Rev.

[6] Dodwell D, Clements K, Lawrence G (2007). Radiotherapy following breast-conserving surgery for screen-detected ductal carcinoma in situ: indications and utilisation in the UK. Interim findings from the Sloane Project. Br J Cancer.

[7] Fisher B, Dignam J, Wolmark N (1999). Tamoxifen in treatment of intraductal breast cancer: National Surgical Adjuvant Breast and Bowel Project B-24 randomised controlled trial. Lancet.

[8] Cuzick J, Sestak I, Pinder SE (2011). Effect of tamoxifen and radiotherapy in women with locally excised ductal carcinoma in situ: long-term results from the UK/ANZ DCIS trial. Lancet Oncol.

[9] Allred DC, Anderson SJ, Paik S (2012). Adjuvant tamoxifen reduces subsequent breast cancer in women with estrogen receptor-positive ductal carcinoma in situ: a study based on NSABP protocol B-24. J Clin Oncol.

[10] Margolese RG, Cecchini RS, Julian TB (2015). Anastrozole versus tamoxifen in postmenopausal women with ductal carcinoma in situ undergoing lumpectomy plus radiotherapy (NSABP B-35): a randomised, double-blind, phase 3 clinical trial. Lancet.

[11] Cuzick J, Sestak I, Forbes JF (2014). Anastrozole for prevention of breast cancer in high-risk postmenopausal women (IBIS-II): an international, double-blind, randomised placebo-controlled trial. Lancet.

[12] Fisher B, Costantino JP, Wickerham DL (1998). Tamoxifen for prevention of breast cancer: report of the National Surgical Adjuvant Breast and Bowel Project P-1 Study. J Natl Cancer Inst.

[13] Cuzick J, Warwick J, Pinney E (2011). Tamoxifen-induced reduction in mammographic density and breast cancer risk reduction: a nested case-control study. J Natl Cancer Inst.

[14] Cox D (1984). Analysis of survival data.

[15] Cox DR (1972). Regression models and life tables. J R Stat Soc.

[16] Kaplan EL, Meier P (1958). Nonparametric estimation from incomplete observations. J Am Stat Assoc.

[17] Cuzick J, Sestak I, Baum M (2010). Effect of anastrozole and tamoxifen as adjuvant treatment for early-stage breast cancer: 10-year analysis of the ATAC trial. Lancet Oncol.

[18] Buzdar A, Howell A, Cuzick J (2006). Comprehensive side-effect profile of anastrozole and tamoxifen as adjuvant treatment for early-stage breast cancer: long-term safety analysis of the ATAC trial. Lancet Oncol.

[19] Dowsett M, Allred C, Knox J (2008). Relationship between quantitative estrogen and progesterone receptor expression and human epidermal growth factor receptor 2 (HER-2) status with recurrence in the Arimidex, Tamoxifen, Alone or in Combination trial. J Clin Oncol.

[20] Cuzick J, Sestak I, Cawthorn S (2015). Tamoxifen for prevention of breast cancer: extended long-term follow-up of the IBIS-I breast cancer prevention trial. Lancet Oncol.

[21] Powles TJ, Ashley S, Tidy A, Smith IE, Dowsett M (2007). Twenty-year follow-up of the Royal Marsden randomized, double-blinded tamoxifen breast cancer prevention trial. J Natl Cancer Inst.

[22] Fisher B, Costantino JP, Redmond CK (1994). Endometrial cancer in tamoxifen-treated breast cancer patients: findings from the National Surgical Adjuvant Breast and Bowel Project (NSABP) B-14. J Natl Cancer Inst.

[23] Early Breast Cancer Trialists' Collaborative Group (EBCTCG) (2005). Effects of chemotherapy and hormonal therapy for early breast cancer on recurrence and 15-year survival: an overview of the randomised trials. Lancet.

[24] Brown SB, Hankinson SE (2015). Endogenous estrogens and the risk of breast, endometrial, and ovarian cancers. Steroids.

[25] Collaborative Group On Epidemiological Studies Of Ovarian Cancer (2015). Menopausal hormone use and ovarian cancer risk: individual participant meta-analysis of 52 epidemiological studies. Lancet.

[26] Rennert G, Rennert HS, Pinchev M, Lavie O, Gruber SB (2009). Use of hormone replacement therapy and the risk of colorectal cancer. J Clin Oncol.

