# Supplementary material for: Anastrozole versus tamoxifen for the prevention of locoregional and contralateral breast cancer in postmenopausal women with locally excised ductal carcinoma in situ (IBIS-II DCIS): a double-blind, randomised controlled trial
Source: Lancet. 2016 Feb 27;387(10021):866–73. doi: 10.1016/S0140-6736(15)01129-0 (PMC4769326; doi:10.1016/S0140-6736(15)01129-0)
Supplement: Supplementary appendix [file mmc1.pdf]

THE LANCET

Supplementary appendix

This appendix formed part of the original submission and has been peer reviewed. We post it as supplied by the authors.

Supplement to: Forbes JF, Sestak I, Howell A, et al, on behalf of the IBIS-II investigators. Anastrozole versus tamoxifen for the prevention of locoregional and contralateral breast cancer in postmenopausal women with locally excised ductal carcinoma in-situ (IBIS-II DCIS): a double-blind, randomised controlled trial. *Lancet* 2015; published online Dec 11. http://dx.doi.org/10.1016/S0140-6736(15)01129-0.

Appendix

Independent Trial Steering Committee
Richard Sainsbury (University College London, United Kingdom)
Judy Garber (Dana-Farber Cancer Institute, Boston, United States)
Jane Warwick (The University of Warwick, Coventry, United Kingdom)

Trial Participant Steering Committee
Bernardo Bonanni (European Institute of Oncology, Milan, Italy National Coordinating Centre)
Mary Buchanan (Patient Advocate)
Nigel Bundred (University Hospital of South Manchester, United Kingdom)
Katharina Buser (Swiss Group for Clinical Cancer Research, Switzerland National Coordinating Centre)
Simon Cawthorn (Southmead Hospital, Bristol)
Robert Coleman (Weston Park Hospital, Sheffield)
Jack Cuzick – Co-Chairman (Queen Mary University, London)
Mitch Dowsett (The Royal Marsden NHS Trust, London)
Richard Eastell (Sheffield University, Sheffield)
Bent Ejlertsen (The Finsen Centre, Copenhagen, Denmark)
Ian Ellis (University of Nottingham)
John Forbes (Australian New Zealand Breast Cancer Trials Group, Newcastle, Australia National Coordinating Centre)
Anthony Howell (Genesis Breast Cancer Prevention Centre, Manchester)
Zsuzsanna Kahan (University of Szeged, Szeged, Hungary)
Christelle Levy (Centre François Baclesse, CAEN & UNICANCER BREAST GROUP, France National Coordinating Centre)
Robert Mansel (University of Wales College of Medicine, Cardiff)
Patrick Neven (UZ Gasthuisberg Ziekenhuis, Leuven, Belgium National Coordinating Centre)
Tiina Palva (Pirkanmaa Cancer Society, Tampere, Finland)
Lisa Rydén (Skånes universitetssjukhus, Lund, Sweden National Coordinating Centre)
Michael Stierer (Austrian Breast and Colorectal Cancer Study Group, Vienna, Austria National Coordinating Centre)
Mary Stuart (Astrazeneca, Macclesfield)
Fatima Vaz (Instituto Portugues de Oncologia, Lisbon, Portugal)
Gunter von Minckwitz (German Breast Group, Frankfurt, Germany National Coordinating Centre)

Principal Investigators

Hugo Marsiglia (Gustave Roussy, Villejuif, UCBG, France)
Christelle Levy (C. F. Baclesse, Caen, UCBG, France)
Ahmed Benyoucef (C. H. Becquerel, Rouen, UCBG, France)
Dominique Berton-Rigaud (C. R. Gauducheau, Saint Herblain, UCBG, France)
Catherine Loustalot (C. G.F. Leclerc, Dijon, UCBG, France)
Daniel Serin (I. Sainte-Catherine, Avignon, UCBG, France)
Pierre Kerbrat (C. E. Marquis, Rennes, UCBG, France)
Jean-Christophe Eymard (I. J. Godinot, Reims, UCBG, France)
Sylvia Giard-Lefevre (C. O. Lambret, Lille, UCBG, France)
Nathalie Bonichon-Lamichhane (Clinique Tivoli, Bordeaux, UCBG, France)
Alain Monnier (CHG, Montbéliard, UCBG, France)
Nicole Tubiana-Mathieu (CHRU Dupuytren, Limoges, UCBG, France)
Gilles Piot (C.M.C. Les Ormeaux, Le Havre, UCBG, France)
Christine Tunon de Lara (Institut Bergonié, Bordeaux, UCBG, France)
Olivier Bernard (CROMG, Agen, UCBG, France)
Hélène Simon-Swirski (CHU A. Morvan, Brest, UCBG, France)
Laurence Gladieff (I. Claudius Regaud, Toulouse, UCBG, France)

Marie-Joseph Fouchet-Goudier (CH Bretagne-Sud, Lorient, UCBG, France)
Nadine Dohollou (PolyClinique Nord Aquitaine, Bordeaux, UCBG, France)
Anne-Marie Elgard-Maitre (CH E. Müller, Mulhouse, UCBG, France)
Jean-Pierre Bergerat (CHRU, Strasbourg, UCBG, France)
Thierry Petit (Centre Paul Strauss, Strasbourg, UCBG, France)
Adina Marti (Centre Hospitalier, Auxerre, UCBG, France)
Caroline Toussaint (Centre Hospitalier, Lagny sur Marne, UCBG, France)
Francesco Del Piano (Hôpitaux du Léman, Thonon les bains, UCBG, France)
Mahmoud Ibrahim (CHR La Source, Orléans, UCBG, France)
Danièle Fric (Institut Daniel Hollard, Grenoble, UCBG, France)
Arnd Hönig (St. Vincenzkrankenhaus Mainz, Mainz, Germany)
Volkmar Müller (Universitätsklinikum Hamburg-Eppendorf, Hamburg, Germany)
Frederik Marmé (Universitätsklinikum Heidelberg, NCT, Heidelberg, Germany)
Beate Rautenberg (Uniklinik Freiburg, Freiburg, Germany)
Marcus Schmidt (Universitätsklinikum Mainz, Mainz, Germany)
Claus Hanusch (Frauenklinik vom Roten Kreuz, München, Germany)
Stefan Paepke (Klinikum rechts der Isar München, München, Germany)
Gabriele Kaltenecker (Städt. Klinikum Karlsruhe, Karlsruhe, Germany)
Sabine Lemster (Klinikum Schaumburg, Stadthagen, Stadthagen, Germany)
Joke Tio (Universitätsklinikum, Münster, Germany)
Toralf Reimer (Universitätsklinikum Rostock, Rostock, Germany)
Benjamin Schnappauf (Universitätsklinikum, Frankfurt/ Main, Germany)
Klaus Baumann (Universitätsklinikum, Marburg, Germany)
Iris Schrader (Henriettenstiftung, Hannover, Germany)
Christoph Mundhenke (Universitätsklinikum Schleswig-Holstein, Campus Kiel, Kiel, Germany)
Cornelia Liedtke (Universitätsklinikum, Lübeck, Germany)
Wolfgang Meinerz (St. Vincenz Krankenhaus GmbH, Paderborn, Germany)
Christoph Thomssen (Klinikum der Med. Fakultät, Halle/Saale, Germany)
Klaus Christl (Krankenhaus Eggenfelden, Eggenfelden, Germany)
Thomas Hitschold (Klinikum Worms gGmbH, Worms, Germany)
Kay Goerke (Mathias-Spital, Rheine, Germany)
Karin Kast (Universitätsklinikum Carl Gistav Carus an der Technischen Universität Dresden, Dresden, Germany)
Ingo Runnebaum (Universitätsklinikum Jena, Jena, Germany)
Christoph Lindner (AGAPLESION DIAKONIEKLINIKUM HAMBURG, Hamburg, Germany)
Peter Scheidel (Marienkrankenhaus Hamburg, Hamburg, Germany)
Uwe Herwig (Albertinen-Krankenhaus, Hamburg, Germany)
Nikola Bangemann (Charité Mitte-Campus Benjamin Franklin, Berlin, Germany)
Harald Sommer (I. Universitäts-Frauenklinik, München, Germany)
Kornelia Göhring (Klinikum Aschaffenburg, Aschaffenburg, Germany)
Augustinus Tulusan (Klinikum Bayreuth, Bayreuth, Germany)
Eva Stauß (Universitätsklinikum, Tübingen, Germany)
Günter Köhler (Universitätsmedizin Greifswald, Greifswald, Germany)
Dirk Zahm (Brustzentrum Ostthüringen am SRH Wald-Klinikum Gera, Gera, Germany)
Doris Augustin (Klinikum Deggendorf, Deggendorf, Germany)
Andrea Hocke (Universitätsfrauenklinik Bonn, Bonn, Germany)
Tanja Neunhöffer (Dr.-Horst-Schmidt-Kliniken GmbH, Wiesbaden, Germany)
Sabine Schmatloch (Elisabeth Krankenhaus GmbH, Kassel, Germany)
Georg Heinrich (Praxis Dr. Heinrich, Fürstenwalde, Germany)
Sabine Groß (Marienhospital, Stuttgart, Germany)
G.P. Breitbach (Marienhausklinik St. Josef-Kohlhof, Neunkirchen, Germany)
Anton Scharl (Klinikum St. Marien Amberg, Amberg, Germany)
Peter Klare (Praxisklinik Krebsheilkunde für Frauen, Berlin, Germany)
Andrea Stefek (Johanniter-Krankenhaus der Altmark, Stendal, Germany)
Gerald Hoffmann (St. Josefshospital, Wiesbaden, Germany)

Franca Martignoni (Uniklinikum Duesseldorf, Duesseldorf, Germany)
Erich Weiss (Klinikum Sindelfingen-Böblingen gGmbH, Böblingen, Germany)
Günter Emons (Universitätsklinikum, Göttingen, Germany)
Hans-Christian Tesch (Onkologie Bethanien / Marien-KHS, Frankfurt, Germany)
Matthias Beckmann (Friedrich-Alexander-Universität, Erlangen, Germany)
Rita Schmutzler (Universitätsklinikum Köln, Koeln, Germany)
Martin Schütte (Katholische Kliniken Essen-Nord, Essen, Germany)
Bahriye Aktas (Universitätsklinikum, Essen, Germany)
Ursula Hille-Betz (Medizinische Hochschule, Hannover, Germany)
Mustafa Deryal (Caritasklinik St. Theresia, Saarbrücken, Germany)
Serban Dan Costa (Otto-v.-Guericke-Universität, Magdeburg, Germany)
Beate Blümel (Onkologische Schwerpunktpraxis, Magdeburg, Germany)
Uwe-Jochen Göhring (Johanniter-Krankenhaus, Bonn, Germany)
Anke Kleine-Tebbe (DRK-Kliniken Berlin Köpenick, Berlin, Germany)
Claudia Schumacher (St.Elisabeth-KKH, Koeln, Germany)
Jasmin/Christoph Pourfard/Uleer (Gemeinschaftspraxis Hildesheim, Hildesheim, Germany)
Uwe Kullmer (Asklepios Paulinen Klinik, Wiesbaden, Germany)
Petra Krabisch (Klinikum Chemnitz, Chemnitz, Germany)
Regine Gätje (Alfried-Krupp Krankenhaus, Essen, Germany)
Thomas Schwenzer (Klinikum Dortmund, Dortmund, Germany)
Hans-Joachim Hindenburg (Praxis Dr. Hindenburg, Berlin, Germany)
Andreas Rempen (Diakonie-Klinikum Schwäbisch HallgGmbH, Schwäbisch Hall, Germany)
Heinz-Gert Höffkes (Klinikum Fulda, Fulda, Germany)
Elmar Stickeler (Brustzentrum Südbaden, Freiburg, Germany)
Christian Reffert (Westpfalz-Klinikum GmbH, Kaiserslautern, Germany)
Stephan Seitz (Caritas-Krankenhaus St. Josef, Regensburg, Germany)
Gerd Splitt (Facharzt f. Frauenheilkunde u. Geburtshilfe, Güstrow, Germany)
Petra Böhne (Pius-Hospital Oldenburg, Oldenburg, Germany)
Karsten Gnauert (Ostalbklinikum, Aalen, Germany)
Hans-Joachim Strittmatter (Kreiskrankenhaus Schorndorf, Schorndorf, Germany)
Gerold Baake (Onkologische Gemeinschaftspraxis, Pinneberg, Germany)
Mahdi Rezai (Luisenkrankenhaus Duesseldorf, Duesseldorf, Germany)
Thomas Noesselt (Sana Klinikum Hameln-Pyrmont, Hameln, Germany)
C.-H. Köhne (Klinikum Oldenburg, Oldenburg, Germany)
Volker Hanf (Klinikum Fürth, Fürth, Germany)
Dirk Strumberg (Marienhospital, Herne, Germany)
Peter Dall (Klinikum Lüneburg, Lüneburg, Germany)
Peter/Bernd Schleicher (Gemeinschaftspraxis Dr. Bern Schleicher/ Peter Schleicher, Schwandorf, Germany)
Frank Beldermann (Karl Olga KKH, Stuttgart, Germany)
Michael Berghorn (Allgemeines Krankenhaus Celle, Celle, Germany)
Dirk-Thoralf Baerens (Frauenarztpraxis, Ilsede, Germany)
Hans-Christian Kolberg (Marienhospital, Bottrop, Germany)
Lelia Bauer (Krankenhaus Weinheim, Weinheim, Germany)
Cosima Brucker (Klinikum Nürnberg, Nürnberg, Germany)
Iris Schrader (Gynäkologisch, onkologische Gemeinschaftspraxis, Hannover, Germany)
Thomas Steck (Klinikum Passau, Passau, Germany)
Frances Boyle (Mater Hospital, North Sydney, Australia)
John Forbes (Calvary Mater Newcastle, Waratah, Australia)
Anupam Chaudhuri (Riverina Cancer Care Centre, Wagga Wagga, Australia)
Eugene Moylan (Liverpool Hospital, Liverpool, Australia)
Jenny Donovan (Royal North Shore Hospital, St. Leonards, Australia)
Stephen Della-Fiorentina (Southern Highlands Cancer Centre, Bowral, Australia)
Ehtesham Abdi (The Tweed Hospital, Tweed Heads, Australia)
Gavin Marx (San Clinical Trials Unit, Wahroonga, Australia)

Geoffrey Beadle (Royal Brisbane & Women's Hospital, Herston, Australia)
Michael Donovan (Nambour Hospital, Nambour, Australia)
Ian Bennett (Princess Alexandra Hospital, Woolloongabba, Australia)
Peter Grantley Gill (Royal Adelaide Hospital, Adelaide, Australia)
Caroline Baker (Austin Heath, Heidelberg, Australia)
Richard Masters (Box Hill Hospital, Box Hill, Australia)
Robert Blum (The Bendigo Hospital, Bendigo, Australia)
John Collins (Royal Melbourne Hospital, Parkville, Australia)
Michael Law (Maroondah Hospital, Ringwood East, Australia)
Stewart Hart (Monash Medical Centre, East Bentleigh, Australia)
Caroline Baker (Victorian Breast & Oncology Care, East Melbourne, Australia)
George Kannourakis (Ballarat Oncology & Haematology Services, Wendouree, Australia)
Raymond Snyder (St Vincent's Hospital, Fitzroy, Australia)
David Joseph (Sir Charles Gairdner Hospital, Nedlands, Australia)
Michael McCrystal (North Shore Hospital (Waitemata), Auckland, New Zealand)
Ian Campbell (Waikato Hospital, Hamilton, New Zealand)
Raimund Jakesz (Vienna University Medical School, Vienna, Austria)
Ursula Selim (Brustzentrum Hanusch-KH, Vienna, Austria)
Christian Singer (Medical University of Vienna, Vienna, Austria)
Dietmar Heck (KH BHS Linz, Linz, Austria)
Richard Greil (St. Johanns Hospital, Salzburg, Austria)
Angela Ramoni (Innsbruck Univ. Medical School, Innsbruck, Austria)
Vesna Bjelic-Radisic (Graz Univ. Med. School, Graz, Austria)
Arno Reichenauer (BHB Hospital Sankt Veit, Sankt Veit, Austria)
Wilfried Horvath (Gussing Hospital, Gussing, Austria)
Josef Thaler (Hospital Klinikum Kreuzschwestern Wels, Oberösterreich, Austria)
Michael Fridrik (General Hospital Linz, Linz, Austria)
Joerg Keckstein (LKH Villach, Villach, Austria)
Lamote Jan (AZ-VUB, Borstkliniek, Brussels, Belgium)
Marc L'Hermitte (CHU-UCV Brugmann, Brussels, Belgium)
Heidi Roelstrate (OLVrouwziekenhuis Aalst, Aalst, Belgium)
Luc Dirix (AZ St. Augustinus, Wilrijk, Belgium)
Inneke Bambust (AZ St. Blasius, Dienst Oncologie, Dendermonde, Belgium)
Monique Seret (St. Elisabeth ZH, Namur, Belgium)
Fabienne Liebens (CHU St. Pierre, Brussels, Belgium)
Maria Maerevoet (Clinque St. Pierre, Ottignies, Belgium)
Lionel D'Hondt (CHU Dinant Godinne, Yvoir, Belgium)
Martine Berliere (Université Catholique de Louvain, Brussels, Belgium)
Jean-Marie Nogaret (Institut Jules Bordet, Brussels, Belgium)
Phillipe Simon (Erasmus ZH, Brussels, Belgium)
Tiina Palva (Pirkanmaa Cancer Society, Tampere, Finland)
Zsuzsanna Kahan (Department of Oncotherapy, University of Szeged, Szeged, Hungary)
Deirdre O'Hanlon (Dept of Surgery, Cork, Ireland)
Henry Paul Redmond (Department of surgery, Cork, Ireland)
Arnold Hill (Surgical Professorial Unit, Beaumont Dublin, Ireland)
Denis Evoy (Department of Surgery, UC Dublin, Ireland)
Michael Kerin (University College Hospital, Galway, Ireland)
Rajnish Gupta (Midwestern Cancer Centre, Limerick, Ireland)
Michael J. Martin (Sligo General Hospital, Sligo, Ireland)
Marcela Fritis (Fundacion Arturo Lopez Perez, Santiago, Chile)
Ricardo Schwartz (Hospital Militar, Santiago, Chile)
Maria Loreto Yañez (Instituto de Radiomedicina, Santiago, Chile)
Octavio Peralta (Hospital Clinico San Borja Arriarán, Santiago, Chile)
Claudio Graiff (Azienda Sanitaria di Bolzano, Bolzano, Italy)
Fabrizio Artioli (Ospedale B. Ramazzini, V. Guido Molinari, Modena, Italy)

Daniele Generali (Azienda Istituti Ospitalieri di Cremona, Cremona, Italy)
Lorenzo Orzalesi (Universita degli studi Firenze, Florence, Italy)
Marilena Visini (Azienda Ospedaliera Ospedale di Lecco, Lecco, Italy)
Bernardo Bonanni (Instituto Europeo di Oncologia, Milan, Italy)
Maria Michiara (Azienda Ospedaliero-Universitaria di Parma, Parma, Italy)
Lorenzo Pavesi (Fondazione Salvatore Maugeri, Pavia, Italy)
Alberto Ravaioli (Azienda Unita Sanitaria Locale di Rimini, Rimini, Italy)
Mauro Porpiglia (Aso O.I.R.M. Sant'anna, Turin, Italy)
Fabio Puglisi (Policlinico Universitario di Udine, Udine, Italy)
Graziella Pinotti (Ospedale di Circolo, Varese, Italy)
Stephen Brincat (Sir Anthony Mamo Oncology Centre, Tal Qroqq, Malta)
Katharina S. Buser (Oncocare Klinik Engeried, Bern, Switzerland)
Manuela Rabaglio (Klinik und Poliklinik fuer Medizinische Onkologie, Inselspital, Bern, Switzerland)
Daniel Rauch (SpitalSTS AG Simmenthal-Thun-Saanenland, Thun, Switzerland)
Pierre O. Chappuis (HUG Unite d'oncogénétique et de prévention, Service d'Oncologie, Genève, Switzerland)
Khalil Zaman (Centre pluridisciplinaire d‘oncologie, CHUV, Lausanne, Switzerland)
Susanne Bucher (Neue Frauenklinik, Luzern, Switzerland)
Barbara Bolliger (Center for Tumordetection and Prevention (ZETUP), St. Gallen, Switzerland)
Olivia Pagani (Institute of Oncology of Southern Switzerland, Bellinzona, Switzerland)
Anna-Karin Falck (Helsingborg Lasarett Helsingborg, Sweden)
Lisa Rydén (Skånes Universitetssjukhus, Lund, Sweden)
Jakob Kaij (Skånes Universitetssjukhu, Malmo, Sweden)
Sara Margolin (Karolinska University Hospital at Södersjukhuset, Stockholm, Sweden)
Mahmut Muslumanoglum (Istanbul University, Istanbul, Turkey)
Gianfilippo Bertelli (Singleton Hospital, Swansea, United Kingdom)
Maria Bramley (The Royal Oldham Hospital, United Kingdom)
James Bristol (Cheltenham General Hospital, United Kingdom)
Sankaran Chandrasekharan (Essex County Hospital, Colchester, United Kingdom)
Perric Crellin (Dorchester County Hospital, United Kingdom)
Raouf Daoud (Frimley Park Hospital NHS Trust, United Kingdom)
David Dodwell (York Hospital, United Kingdom)
Philip Drew (Royal Cornwall Hospital, Truro, United Kingdom)
Sidharth Dubey (Derriford Hospital, Devon, United Kingdom)
Abigail Evans (Poole Hospital NHS Foundation Trust, United Kingdom)
Douglas Ferguson (Royal Devon and Exeter Hospital, United Kingdom)
Raafat Gendy (Mid Staffordshire NHS Foundation Trust, United Kingdom)
Hisham Hamed (Guys Hospital, London, United Kingdom)
Claudia Harding-McKean (Countess of Chester Hospital, United Kingdom)
Chris Holcombe (Royal Liverpool University Hospital, United Kingdom)
Kieran Horgan (St James' Hospital, Leeds, United Kingdom)
Shabana Iqbal (Huddersfield Royal Infirmary, United Kingdom)
Jibril A. Jibril (United Lincolnshire Hospitals NHS Trust, United Kingdom)
Jalal Kokan (Macclesfield District General Hospital, United Kingdom)
Peter Kneeshaw (The Hull and East Yorkshire Breast Care Unit, United Kingdom)
Mark Lansdown (St James' Hospital, Leeds, United Kingdom)
Tom Lennard (University of Newcastle upon Tyne, United Kingdom)
Rick Linforth (St Lukes Hospital, Bradford, United Kingdom)
Robert Mansel (University of Wales College of Medicine, Cardiff, United Kingdom)
Stuart McIntosh (Belfast City Hospital, United Kingdom)
Sankha Mitra (Worthing and Southlands Hospital, United Kingdom)
Glyn Neades (Western General Hospital, Edinburgh, United Kingdom)
Jane Louise Ooi (Royal Bolton Hospital, United Kingdom)
Ashraf Patel (St. Margaret's Hospital, Epping, United Kingdom)
Zenon Rayter (Bristol Royal Infirmary, United Kingdom)

Robert Reichert (Northwick Park Hospital, Harrow, United Kingdom)
Fiona Roberts (St. James's University Hospital, Wakefield, United Kingdom)
Nicola Roche (Royal Marsden Hospital, London, United Kingdom)
Colin Rogers (Queens Hospital Burton, Burton-on-Trent, United Kingdom)
Gavin Royle (Princess Anne Hospital, Southampton, United Kingdom)
Elizabeth Shah (East Sussex Hospitals NHS Trust, United Kingdom)
Mark Sibbering (Royal Derby Hospital, United Kingdom)
Anthony Iain Skene (Royal Bournemouth Hospital, United Kingdom)
Simon Smith (Broomfield Hospital, Chelmsford, United Kingdom)
Geoffrey Sparrow (Yeovil District Hospital, Yeovil, United Kingdom)
Alastair Thompson (Ninewells Hospital, Dundee, United Kingdom)
Jayant Vaidya (Royal Free and UCL Medical School, London, United Kingdom)
Virginia Wolstenholme (St Bartholomew's Hospital, London, United Kingdom)
Jeremy Wood (Queen Elizabeth II Hospital, Welwyn Garden City, United Kingdom)
Constantinos Yiangou (Queen Alexandra Hospital, Portsmouth, United Kingdom)
Charles Zammit (Royal Sussex County Hospital, Brighton, United Kingdom)

Local Coordinating Centres

Rochelle Thornton (Australian New Zealand Breast Cancer Trials group, Newcastle, Australia)
Flonda Probert (Australian New Zealand Breast Cancer Trials group, Newcastle, Australia)
Akiko Fong (Australian New Zealand Breast Cancer Trials group, Newcastle, Australia)
Nicole Francis (Australian New Zealand Breast Cancer Trials group, Newcastle, Australia)
Manuela Gili (Austrian Breast and Colorectal Cancer Study Group, Vienna, Austria)
Rosita Eigenberger (Austrian Breast and Colorectal Cancer Study Group, Vienna, Austria)
Daisy Supply (University Hospitals, Leuven, Belgium)
Inge Lefever (University Hospitals, Leuven, Belgium)
Bettina Muller (Chilean Cooperative Group for Oncologic Research (GOCCHI), Santiago, Chile)
Zdenka Zlatar (Chilean Cooperative Group for Oncologic Research (GOCCHI), Santiago, Chile)
Petra Feer (GBG Forschungs GMBH, Frankfurt, Germany)
Ioannis Gkantiragas (GBG Forschungs GMBH, Frankfurt, Germany)
Marjo Virkki (Tampere, Tampere, Finland)
Sibille Everhard (La Fédération Nationale des Centres de Lutte Contre le Cancer, Paris, France)
Jerome Lemonnier (La Fédération Nationale des Centres de Lutte Contre le Cancer, Paris, France)
Sara Garcia (La Fédération Nationale des Centres de Lutte Contre le Cancer, Paris, France)
Saliha Ghanem (La Fédération Nationale des Centres de Lutte Contre le Cancer, Paris, France)
Anna Cole (All Ireland Cooperative Clinical Research Group (ICORG), Cork, Ireland)
Debra O'Hare (All Ireland Cooperative Clinical Research Group (ICORG), Cork, Ireland)
Elaine Cronin (All Ireland Cooperative Clinical Research Group (ICORG), Cork, Ireland)
Trudi Roche (All Ireland Cooperative Clinical Research Group (ICORG), Dublin, Ireland)
Emer Kennedy (All Ireland Cooperative Clinical Research Group (ICORG), Dublin, Ireland)
Jo Ballot (All Ireland Cooperative Clinical Research Group (ICORG), Dublin, Ireland)
Niamh Killilea (All Ireland Cooperative Clinical Research Group (ICORG), Galway, Ireland)
Marian Jennings (All Ireland Cooperative Clinical Research Group (ICORG), Galway, Ireland)
Laura Lowry (All Ireland Cooperative Clinical Research Group (ICORG), Limerick, Ireland)
Moira Maxwell (All Ireland Cooperative Clinical Research Group (ICORG), Sligo, Ireland)
Margaret Burke (All Ireland Cooperative Clinical Research Group (ICORG), Sligo, Ireland)
Aliana Guerrieri Gonzaga (Istituto Europeo di Oncologia, Milan, Italy)
Giorgia Bollani (Istituto Europeo di Oncologia, Milan, Italy)
Andrea Bianchetti (A.O. Papa Giovanni XXIII, Bergamo, Italy)
Anna Scalvini (A.O. Spedali Civili di Brescia, Brescia, Italy)
Elisabetta Cretella (Azienda Sanitaria di Bolzano, Bolzano, Italy)
Antonella Pasqualini (Ospedale B. Ramazzini, Carpi, Italy)
Angela Gobbi (Az. Istituti Ospitalieri di Cremona, Cremona, Italy)
Jenny Roselli (Policlinico Careggi Università di Firenze, Florence, Italy)

Angelita Lagati (Azienda Ospedaliera Ospedale di Lecco, Lecco, Italy)
Elena Rapacchi (Azienda Ospedaliero-Universitaria di Parma, Parma, Italy)
Annalisa Lanza (Fondazione Salvatore Maugeri, Pavia, Italy)
Emanuela Pini (Azienda USL della Romagna Ospedale Infermi Rimini, Rimini, Italy)
Elisa Picardo (Azienda Ospedaleiera Sant'Anna, Turin, Italy)
Furio Maggiorotto (Istituto per la Ricerca e Cura del Cancro, Candiolo (TO), Italy)
Roberta Sottile (Policlinico Universitario di Udine, Udine, Italy)
Ilaria Vallini (Ospedale di Circolo e Fondazione Macchi, Varese, Italy)
Nadia Cilia (Sir Anthony Mamo Oncology Centre, Tal Qroqq, Malta)
Mital Patel (Barts Hospital, London, United Kingdom)
Linda Bamford (Bradford Royal Infirmary, Bradford, United Kingdom)
Helen Robertshaw (Bradford Royal Infirmary, Bradford, United Kingdom)
Hayle Inman (Bradford Royal Infirmary, Bradford, United Kingdom)
Naomi Hill (Belfast City Hospital, Belfast, United Kingdom)
Jane Dexter (Royal Sussex County Hospital Brighton, United Kingdom)
Emily Peasgood (Royal Sussex County Hospital, Brighton, United Kingdom)
Imogen Batty (Royal Bournemouth Hospital, Bournemouth, United Kingdom)
Shirley Cocks (Royal Bolton Hospital, Bolton, United Kingdom)
Raksha Mistry (Royal Bolton Hospital, Bolton, United Kingdom)
Mary Sidders (Bristol Royal Infirmary, Bristol, United Kingdom)
Emily Foulstone (Bristol Royal Infirmary, Bristol, United Kingdom)
Helen Garlicka (Southmead Hospital, Bristol, United Kingdom)
Catherine Dawe (Southmead Hospital, Bristol, United Kingdom)
Jackie Elliott (Queens Hospital Burton, Burton-on-Trent, United Kingdom)
Kathy Rooke (Essex County Hospital, Colchester, United Kingdom)
Christine Morris (University Hospital of Llandough, Cardiff, United Kingdom)
Yvonne Lester (Mid Essex Hospital Services NHS Trust, Chelmsford, United Kingdom)
Sian Gibson (Mid Essex Hospital Services NHS Trust, Chelmsford, United Kingdom)
Jill Chittock (Cheltenham General Hospital, Cheltenham, United Kingdom)
Amy Skelton (Cheltenham General Hospital, Cheltenham, United Kingdom)
Elizabeth Gallimore (Countess of Chester Hospital, Chester, United Kingdom)
Charlotte Downes (Royal Derby Hospital, Derby, United Kingdom)
Lynn Billett (Dorchester County Hospital, Dorchester, United Kingdom)
Simone Caddy (Dorchester County Hospital, Dorchester, United Kingdom)
Helen Cumming (Ninewells Hospital, Dundee, United Kingdom)
Sharon Cowell-Smith (Western General, Edinburgh, United Kingdom)
Caroline Turner (Princess Alexandra Hospital, Epping, United Kingdom)
Sunjalee Fernando (Princess Alexandra Hospital, Epping, United Kingdom)
Sarah Goodwin (Conquest Hospital, St Leonards On Sea, United Kingdom)
Jo-Anne Taylor (Conquest Hospital, St Leonards On Sea, United Kingdom)
Kizzy Baines (Royal Devon and Exeter Hospital, Exeter, United Kingdom)
Susan Downer (Royal Devon and Exeter Hospital, Exeter, United Kingdom)
Alice Pilcher (Frimley Park Hospital, Frimley, United Kingdom)
Tracey Dobson (Queen Alexandra Hospital, Portsmouth, United Kingdom)
Lynn Osborne (Grantham and District Hospital, Grantham, United Kingdom)
Greg Kuenzig (Guy's & St Thomas' NHS Foundation Trust, London, United Kingdom)
Denise Hancock (Huddersfield Royal Infirmary, Huddersfield, United Kingdom)
Deborah Melia (Huddersfield Royal Infirmary, Huddersfield, United Kingdom)
Clinical Trials Department Pharmacy (Castle Hill Hospital, Cottingham, United Kingdom)
Elaine Gullaksen (Castle Hill Hospital, Cottingham, United Kingdom)
Susan Hartup (St James's University Hospital, Leeds, United Kingdom)
Amy Henson (St James's University Hospital, Leeds, United Kingdom)
Jane Gibb (St James's University Hospital, Leeds, United Kingdom)
Sarah Coombs (Lincoln County Hospital, Lincoln, United Kingdom)
Caroline Taylor (Lincoln County Hospital, Lincoln, United Kingdom)

Amy Kirkby (Pilgrim Hospital, Lincoln, United Kingdom)
Issy Thomas (Pilgrim Hospital, Lincoln, United Kingdom)
Karen Makinson (Royal Liverpool University Hospital, Liverpool, United Kingdom)
Yukie Kano (Royal Marsden Hospital, London, United Kingdom)
Zoheb Shah (Royal Marsden Hospital, London, United Kingdom)
Lisa Hardstaff (Macclesfield Hospital, Macclesfield, United Kingdom)
Barbara Townley (Macclesfield Hospital, Macclesfield, United Kingdom)
Philippa Hill (Macclesfield Hospital, Macclesfield, United Kingdom)
Marilyn McCurrie (Macclesfield Hospital, Macclesfield, United Kingdom)
Sue Grassby (University Hospital of South Manchester, Manchester, United Kingdom)
Pamela Henderson (Royal Victoria Infirmary, Newcastle-Upon-Tyne, United Kingdom)
Elizabeth Talbot (Royal Victoria Infirmary, Newcastle-Upon-Tyne, United Kingdom)
Ashley Bell (Royal Victoria Infirmary, Newcastle-Upon-Tyne, United Kingdom)
Reshma Kanani (Northwick Park Hospital, Northwick Park, United Kingdom)
Joanne Johnson (Royal Oldham Hospital, Oldham, United Kingdom)
Richard Jones (Royal Oldham Hospital, Oldham, United Kingdom)
Mel Foster (Poole Hospital, Poole, United Kingdom)
Becky Troke (Poole Hospital, Poole, United Kingdom)
Hilary Congdon (Derriford Hospital, Plymouth, United Kingdom)
Julie Pascoe (Derriford Hospital, Plymouth, United Kingdom)
Sian Whelan (Singleton Hospital, Swansea, United Kingdom)
Jenna Edwards (Singleton Hospital, Swansea, United Kingdom)
Elaine Brinkworth (Singleton Hospital, Swansea, United Kingdom)
Sheila Blizard (Weston Park Hospital, Sheffield, United Kingdom)
Alison Clarke (Weston Park Hospital, Sheffield, United Kingdom)
Kim Stevens (Southampton General Hospital, Southampton, United Kingdom)
Carol Harvey (Staffordshire General Hospital, Stafford, United Kingdom)
Jill Stacey (Staffordshire General Hospital, Stafford, United Kingdom)
Sadie Mitchell (Royal Cornwall Hospital, Truro, United Kingdom)
Tracey Lowry (Pinderfields Hospital, Wakefield, United Kingdom)
Sarah Buckley (Pinderfields Hospital, Wakefield, United Kingdom)
Clare Collins (Queen Elizabeth II Hospital, Welwyn Garden, United Kingdom)
Liz Green (Queen Elizabeth II Hospital, Welwyn Garden, United Kingdom)
Helen Jones (Worthing and Southlands Hospital, Worthing, United Kingdom)
Veronica Conteh (Whittington Hospital, Whittington, United Kingdom)
Tracey Ducket (Yeovil District Hospital, Yeovil, United Kingdom)
Michelle Kotze (Yeovil District Hospital, Yeovil, United Kingdom)
Paula Strider (York Hospital, York, United Kingdom)
Marina Stouraitis (SAKK - Swiss Group for Clinical Cancer Research, Bern, Switzerland)
Jan Sundberg (Lund University Hospital, Lund, Sweden)

Independent Data Monitoring Committee
Abdel Babiker (Medical Research Council Clinical Trials Unit, University College London)
Rory Collins (University of Oxford)
Jan Klijn (Erasmus Medical Centre, Rotterdam, Netherlands)
Stuart Ralston (University of Edinburgh)
Martin Tattersall (University of Sydney, Australia)
Ian Weller (University College London)

IBIS-II Coordinating Centre, Queen Mary University of London
Andreia De Sousa
Rob Edwards
Sheila Ferguson
Jane Hickman
Damian Johnson

Nadia Haidar
Victoria Hammond
Emma Heighway
Amalia Ndoutoumou
Navdip Sahota
Laura White

Cancer Research UK Barts Clinical Trials Unit, Cancer Prevention Trials Unit
Benoit Aigret
Priyanka Batra
Jill Knox
Adedayo Oke
Richard Ostler
Peter Sasieni

Supplementary Figure 1: Five year adherence according to treatment allocation.

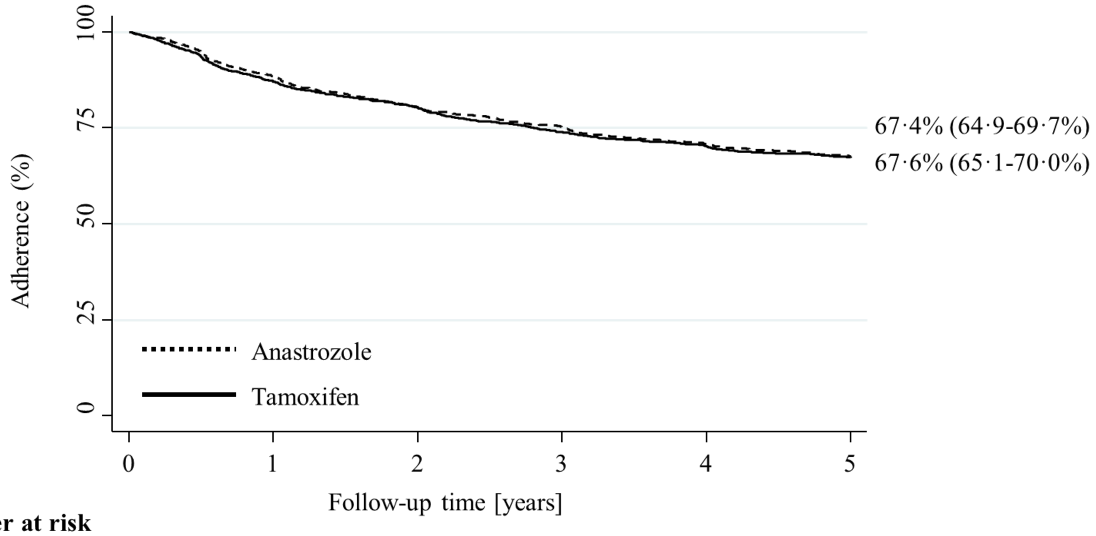

Number at risk

Tamoxifen	1489	1293	1192	1099	1006	862
Anastrozole	1449	1278	1161	1087	980	841

Supplementary Table 1: Cause of death by treatment allocation.

	**Anastrozole (N=1449)**	**Tamoxifen (N=1489)**
**All**	**33 (2·3%)**	**36 (2·4%)**
Breast cancer	1	3
Other cancer	15	12
Colorectal cancer	2	2
Lung cancer	6	2
Endometrial cancer	0	1
Pancreas cancer	1	3
CVA/Stroke/Thromboembolic	5	7
Myocardial infarction	2	2
Other	4	5
Unknown	6	7

*CVA=Cerebrovascular accident
